# Determining the elastography strain ratio cut off value for differentiating benign from malignant breast lesions: systematic review and meta-analysis

**DOI:** 10.1186/s40644-022-00447-5

**Published:** 2022-02-12

**Authors:** Timothy Musila Mutala, Gladys N. Mwango, Angeline Aywak, Dania Cioni, Emanuele Neri

**Affiliations:** 1 Department of Diagnostic Imaging and Radiation Medicine, University of Nairobi, Faculty of Health Sciences, P.O. Box 19676 00502, KNH, Nairobi, Kenya; 2 Department of Translational Research, Master Course in Oncologic Imaging, University of Pisa, Via Roma 67 –, 56124 Pisa, Italy

**Keywords:** Strain elastography, Strain ratio, Cut-off value, Breast masses, Breast cancer imaging

## Abstract

**Background:**

Elastography is an addition to grey-scale ultrasonic examination that has gained substantial traction within the last decade. Strain ratio (SR) has been incorporated as a semiquantitative measure within strain elastography, thus a potential imaging biomarker. The World Federation for Ultrasound in Medicine and Biology (WFUMB) published guidelines in 2015 for breast elastography. These guidelines acknowledge the marked variance in SR cut-off values used in differentiating benign from malignant lesions. The objective of this review was to include more recent evidence and seek to determine the optimal strain ratio cut off value for differentiating between benign and malignant breast lesions.

**Methods:**

Comprehensive search of MEDLINE and Web of Science electronic databases with additional searches via Google Scholar and handsearching set from January 2000 to May 2020 was carried out. For retrieved studies, screening for eligibility, data extraction and analysis was done as per the Preferred Reporting Items for Systematic Reviews and Meta-Analyses for Diagnostic Test Accuracy (PRISMA-DTA) Statement guidelines of 2018. Quality and risk of bias assessment of the studies were performed using the revised Quality Assessment of Diagnostic Accuracy Studies (QUADAS-2) tool.

**Results:**

A total of 424 articles, 412 from electronic database and 12 additional searches were retrieved and 65 studies were included in the narrative synthesis and subgroup analysis. The overall threshold effect indicated significant heterogeneity among the studies with Spearman correlation coefficient of Logit (TPR) vs Logit (FPR) at − 0.301, *p*-value = 0.015. A subgroup under machine model consisting seven studies with 783 patients and 844 lesions showed a favourable threshold, Spearman’s correlation coefficient,0.786 (*p* = 0.036).

**Conclusion:**

From our review, currently the optimal breast SR cut-off point or value remains unresolved despite the WFUMB guidelines of 2015. Machine model as a possible contributor to cut-off value determination was suggested from this review which can be subjected to more industry and multi-center research determination.

**Supplementary Information:**

The online version contains supplementary material available at 10.1186/s40644-022-00447-5.

## Background

Breast cancer is the leading type of cancer both in diagnosis and mortality among women globally [1]. It is also estimated that 627,000 women died from the same in 2018 [2]. Early and accurate diagnosis is essential for its management as well as for the overall well-being of the woman.

Cytology or histology (biopsy) are the definitive diagnostic approach methods. However, imaging has provided a pathway in the diagnosis that reduces unnecessary and invasive cytology or biopsy, a strategy adopted by many guidelines. Breast ultrasound is one of the diagnostic imaging methods with lexicons like BI-RADS being key in differentiating benign from malignant lesions. This has been reported to have a global pooled sensitivity and specificity of 80.1 and 88.4% respectively [3].

Elastography is an addition to grey-scale ultrasonic examination that has gained substantial traction within the last decade. This bases its function on the fact that tissue elasticity can be a predictor of malignancy. Benign lesions are mainly expected to be more elastic while their malignant counterparts will most likely be stiffer. Strain elastography is currently more available than shear wave elastography. Further, strain ratio (SR) has been incorporated as a semiquantitative measure, thus a potential imaging biomarker. The number of articles that are being published annually on breast ultrasound elastography indicate that it is an evolving field. A meta-analytic study of SR carried out in 2012 showed a wide range of cut-off value from 0.5 to 4.5 [4]. However, that review included only nine studies among the ones which were available by then. The World Federation for Ultrasound in Medicine and Biology (WFUMB) published guidelines in 2015 for breast elastography [5, 6]. These guidelines acknowledge the marked variance in SR cut-off values used in differentiating benign from malignant lesions. At the same time, through scanning of literature there are more research papers that have been published since then. For SR to be fully established as a potential imaging biomarker for differentiating between benign and malignant breast lesions a more optimal cut-off value needs to be deduced. It is for that reason that in this review, we intended to determine the most current status in resolving the cut-off value.

The objective of this review was to include more recent evidence and seek to determine the SR cut off value for differentiating between benign and malignant breast lesions.

## Methods

This study’s protocol was not registered or shared with any organization other than the University of Pisa’s Department of Translational Research. We used the Preferred Reporting Items for Systematic Reviews and Meta-Analyses for Diagnostic Test Accuracy (PRISMA-DTA) Statement guidelines of 2018 [7] in carrying out the study and disseminating our findings.

### Search strategy

We did comprehensive search of MEDLINE and Web of Science electronic databases with additional searches via Google Scholar and handsearching mainly through references of articles that were retrieved. The period of search was set from January 2000 to May 2020. Studies of interest were the those that had a patient population of breast mass and/or breast cancer with an index test of ultrasound with elastography and strain ratio calculated. Our search was done using combination of headings and terms that included at least two key words. The key words were “breast mass”, “elastography”, “strain ratio”, “breast ultrasound”, “histology”, “biopsy”, “cytology”, “breast cancer” and “cut off value”. The term “strain ratio” had to appear in each combination set.

### Study selection

Three researchers (TMM, AA and GM) reviewed the retrieved articles and reached consensus on eligible study criteria. The inclusion criteria for the studies were as follows: (a) Breast ultrasound strain elastography with SR calculation performed. (b) Setting where ultrasound elastography examination was done before a reference standard diagnostic test and treatment. (c) Acceptable standard reference test like biopsy or cytology results and/or relevant follow up results of BI-RADS III lesions. (d) Adequate data presented in a format that could lead to creating a diagnostic study 2 × 2 table. (e) Articles in English language. (f) Reporting threshold of 24 “yes” out of 30 responses to the Standards for Reporting of Diagnostic Accuracy Studies (STARD) 2015 criteria [8].

### Data extraction and quality assessment

A data extraction plan was executed in which a total of 31 variables were identified within the realms of participants (patients with breast mass or masses), index test (SR), reference standard (pathological diagnosis) and target condition (malignancy). In addition, general variables of the studies, that is author, year of publication, journal and country were also included.

Quality assessment of the studies was performed using the revised Quality Assessment of Diagnostic Accuracy Studies (QUADAS-2) tool [9]. This entailed assessment of risk of bias as well as the applicability. The observations of the assessment were presented both in tabular and graphic formats.

For each eligible study, a 2 × 2 table depicting true positives, false positives, false negatives and true negatives was constructed. The principal diagnostic accuracy measures were sensitivity and specificity per lesion.

### Data synthesis and statistical analyses

Primary characteristics of the studies were entered as variables using MS Excel® worksheets.

Descriptive statistics from the diagnostic accuracy 2 × 2 tables of each individual study were computed using MedCalc® [10] and Meta-Disc [11] statistical software calculators. The measures were sensitivity, specificity, positive likelihood ratio (PLR), negative likelihood ratio (NLR) and diagnostic odds ratio (DOR).

Forest plots of sensitivity and specificity were deduced using Stata® statistical software [12]. A summary receiver operating characteristic (SROC) curve plot for sensitivity against 1-specificty (false positive rate) was also done using the Stata® software.

Heterogeneity and threshold effect were assessed in several stages as per standard recommendations [13] . First, visual examinations of the coupled forest plots of sensitivity and specificity as well as the SROC plot were done. Secondly, a Spearman’s correlation was calculated for sensitivity vs false positive rate. Lastly, the Cochran’s Q and Higgin’s (I^2^) statistics (though not much weight was given to them) were deduced during the derivation of the forest plots.

Qualitative (narrative) synthesis was done following failure to resolve heterogeneities of most of the studies through meta-regression and subgroup analysis of the studies. The subgroup variables were according to participants characteristics (age, sex and global region), index test (various strain ratio measurement methods) and reference standard approach methods.

Publication bias was interrogated using Funnel plot and Egger’s regression test of DORs against their standards errors (se) with the help of Stata® software.

Meta-analysis was carried out on a single subgroup that showed favourable threshold effects during the sub-group analysis process using Stata® software. A hierarchical summary receiver operating characteristic (HSROC) curve was constructed with the summary points displayed. Youden index was calculated and optimal cut-off strain ratio value for this subgroup derived.

## Results

### Study selection

**Fig. 1:**
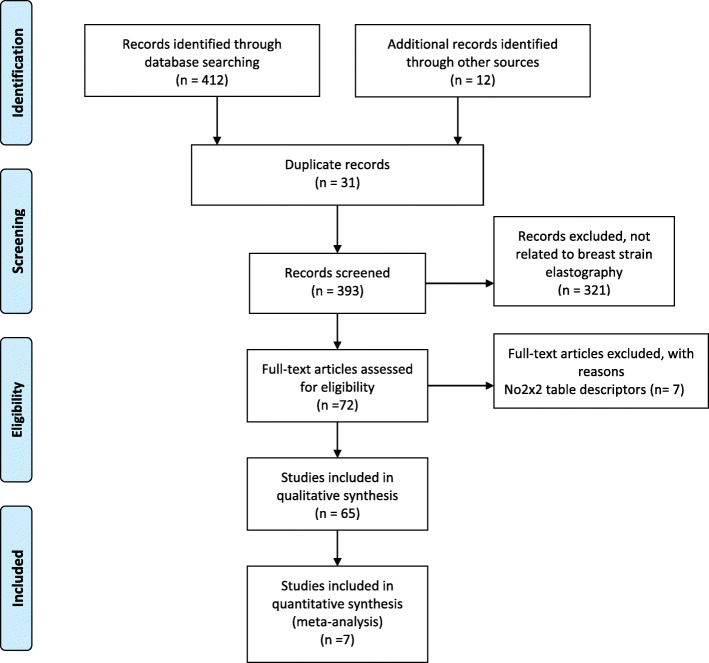
PRISMA flow chart of study selection

A flow chart representing the study selection process is as shown in Fig. 1. A total of 424 articles, 412 from electronic database and 12 additional searches were retrieved. Duplicated articles were 31 and following title and abstract screening, 321 were found not to be related to breast strain elastography. Therefore, 72 full articles that met eligibility criteria were accessed. These had also scored at least 24 out of 30 points in the STARD checklist. Seven of the studies did not have enough data to provide a 2 × 2 table for diagnostic accuracy. Hence, 65 studies were included in the narrative synthesis and subgroup analysis. Meta-analysis was only feasible in one of the subgroups consisting seven studies as the rest had significant between study heterogeneities and unfavourable threshold effects that could not be resolved.

### Study characteristics

An overview of the study characteristics is as shown in Table 1. The total number of lesions included in this systematic review was 11,759 but the total number of patients could not be computed as two authors had only provided the number of lesions in their studies. Malignant and benign lesions were 4897 (41.64%) and 6862 (58.46%) respectively. The combined age range documented was 12 to 93 years with a mean of 56.44 years. In terms of sex, 38 (58.46%) studies included only female patients, three (4.62%) had a mixed male and female population while 24 (36.92%) were not clear on this variable.

**Table 1:** Study characteristics

Study	Continent	Design	Number of Lesions	Setting	Index test (Strain Ratio) Measurement Method	Reference Standard	Target Condition Definition
Ahmed 2020 [14]	Africa	Prospective	132	Imaging before biopsy	FLR	Mixed	Malignant Vs Benign
Alhabshi 2013 [15]	Asia	Prospective	168	Imaging before biopsy	FLR	Mixed	Malignant Vs Benign
Arslan 2018 [16]	Europe	Retrospective	113	Imaging before biopsy	Not Clear	Mixed	Malignant Vs Benign
Balcik 2016 [17]	Europe	Prospective	135	Imaging before biopsy	FLR	Core Biopsy	Malignant Vs Benign
Bojanic 2017 [18]	Europe	Prospective	130	Imaging before biopsy	FLR	Mixed	Malignant Vs Benign
Chee 1 2019 [19]	Asia	Prospective	53	Imaging before biopsy	FLR	Mixed	Malignant Vs Benign
Chee 2 2019 [19]	Asia	Prospective	53	Imaging before biopsy	GLR	Mixed	Malignant Vs Benign
Cheng 2018 [20]	Asia	Prospective	37	Imaging before biopsy	Mixed FLR and GLR	Mixed	Malignant Vs Benign
Cho 2010 [21]	Asia	Prospective	99	Imaging before biopsy	FLR	Mixed	Malignant Vs Benign
Dawood 2018 [22]	Africa	Prospective	40	Imaging before biopsy	FLR	Mixed	Malignant Vs Benign
Fausto 2015 [23]	Europe	Prospective	129	Imaging before biopsy	Mixed FLR and GLR	Core Biopsy	Malignant Vs Benign
Fujioka 2019 [24]	Asia	Retrospective	148	Imaging before biopsy	FLR	Mixed	Malignant Vs Benign
Gheonea 2011 [25]	Europe	Prospective	58	Imaging before biopsy	FLR	Mixed	Malignant Vs Benign
Gong 2013 [26]	Asia	Prospective	192	Imaging before biopsy	Not Clear	Mixed	Malignant Vs Benign
Graziano 1 2017 [27]	South America	Prospective	159	Imaging before biopsy	FLR	Mixed	Malignant Vs Benign
Graziano 2 2017 [27]	South America	Prospective	159	Imaging before biopsy	GLR	Mixed	Malignant Vs Benign
Guruf 2019 [28]	Europe	Prospective	87	Imaging before biopsy	FLR	Core Biopsy	Malignant Vs Benign
Hao 2020 [29]	Asia	Prospective	311	Imaging before biopsy	GLR	Mixed	Malignant Vs Benign
Jung Hahn 2012 [30]	Asia	Retrospective	110	Imaging before biopsy	FLR	Mixed	Malignant Vs Benign
Jung Park 1 2016 [31]	Asia	Prospective	358	Imaging before biopsy	Mixed FLR and GLR	Mixed	Malignant Vs Benign
Jung Park 2 2016 [31]	Asia	Prospective	358	Imaging before biopsy	FLR	Mixed	Malignant Vs Benign
Khamis 2017 [32]	Africa	Prospective	120	Imaging before biopsy	FLR	Mixed	Malignant Vs Benign
Kim Kim 2018 [33]	Asia	Prospective	108	Imaging before biopsy	FLR	Core Biopsy	Malignant Vs Benign
Kumm 2010 [34]	North America	Prospective	310	Imaging before biopsy	FLR	Core Biopsy	Malignant Vs Benign
Lee 2011 [35]	Asia	Retrospective	315	Imaging before biopsy	FLR	Core Biopsy	Malignant Vs Benign
Li Wang 2015 [36]	Asia	Retrospective	89	Imaging before biopsy	Mixed FLR and GLR	Mixed	Malignant Vs Benign
Liu 2014 [37]	Asia	Retrospective	431	Imaging before biopsy	FLR	Core Biopsy	Malignant Vs Benign
Mansour 2012 [38]	Africa	Prospective	97	Imaging before biopsy	Mixed FLR and GLR	Mixed	Malignant Vs Benign
Menezes 2016 [39]	Asia	Prospective	143	Imaging before biopsy	FLR	Mixed	Malignant Vs Benign
Mu 2016 [40]	Asia	Retrospective	1080	Imaging before biopsy	Mixed FLR and GLR	Mixed	Malignant Vs Benign
Mutala 2016 [41]	Africa	Prospective	112	Imaging before biopsy	FLR	Core Biopsy	Malignant Vs Benign
Nakashima 2018 [42]	Asia	Prospective	232	Imaging before biopsy	FLR	Core Biopsy	Malignant Vs Benign
Ozel 2018 [43]	Europe	Prospective	297	Imaging before biopsy	Mixed FLR and GLR	Core Biopsy	Malignant Vs Benign
Ozsoy 2016 [44]	Europe	Prospective	168	Imaging before biopsy	FLR	Core Biopsy	Malignant Vs Benign
Parajuly 2012 [45]	Asia	Prospective	342	Imaging before biopsy	Not Clear	Mixed	Malignant Vs Benign
Park 1 2020 [46]	Asia	Retrospective	140	Imaging before biopsy	FLR	Core Biopsy	Malignant Vs Benign
Park 2 2020 [46]	Asia	Retrospective	140	Imaging before biopsy	FLR	Core Biopsy	Malignant Vs Benign
Park 3 2020 [46]	Asia	Retrospective	140	Imaging before biopsy	FLR	Core Biopsy	Malignant Vs Benign
Rehman 2017 [47]	Asia	Prospective	137	Imaging before biopsy	Not Clear	Core Biopsy	Malignant Vs Benign
Ricci 2017 [48]	Europe	Prospective	242	Imaging before biopsy	FLR	Mixed	Malignant Vs Benign
Seo 2018 [49]	Asia	Prospective	45	Imaging before biopsy	FLR	Mixed	Malignant Vs Benign
Singla 2019 [50]	Asia	Prospective	199	Imaging before biopsy	Mixed FLR and GLR	Mixed	Malignant Vs Benign
Stachs 2013 [51]	Europe	Prospective	224	Imaging before biopsy	Not Clear	Not Clear	Malignant Vs Benign
Stoian 2016 [52]	Europe	Prospective	174	Imaging before biopsy	Not Clear	Surgical	Malignant Vs Benign
Thomas 2010 [53]	Europe	Prospective	227	Imaging before biopsy	FLR	Core Biopsy	Malignant Vs Benign
Turker 2017 [54]	Europe	Prospective	75	Imaging before biopsy	FLR	Core Biopsy	Malignant Vs Benign
Ueno 1 2015[55]	Asia	Prospective	98	Imaging before biopsy	FLR	Core Biopsy	Malignant Vs Benign
Ueno 2 2007 [56]	Asia	Not Clear	406	Imaging before biopsy	Not Clear	Not Clear	Malignant Vs Benign
Wang 2018 [57]	Asia	Prospective	302	Imaging before biopsy	Mixed FLR and GLR	Mixed	Malignant Vs Benign
Yagci 2017 [58]	Europe	Prospective	68	Imaging before biopsy	FLR	Core Biopsy	Malignant Vs Benign
Yerli 2011[59]	Europe	Prospective	78	Imaging before biopsy	Mixed FLR and GLR	Mixed	Malignant Vs Benign
Yildiz 2020 [60]	Europe	Prospective	50	Imaging before biopsy	FLR	Core Biopsy	Malignant Vs Benign
Yilmaz 2017 [61]	Europe	Prospective	79	Imaging before biopsy	FLR	Core Biopsy	Malignant Vs Benign
Yoon 1 2016 [62]	Asia	Prospective	201	Imaging before biopsy	FLR	Mixed	Malignant Vs Benign
Yoon 2 2017 [63]	Asia	Prospective	243	Imaging before biopsy	FLR	Mixed	Malignant Vs Benign
Yoon 3 2017 [63]	Asia	Prospective	243	Imaging before biopsy	FLR	Mixed	Malignant Vs Benign
You 2019 [64]	Asia	Retrospective	373	Imaging before biopsy	FLR	Mixed	Malignant Vs Benign
Youk 2014 [65]	Asia	Retrospective	79	Imaging before biopsy	Not Clear	Mixed	Malignant Vs Benign
Zhang 1 2020 [66]	Asia	Prospective	91	Imaging before biopsy	Mixed FLR and GLR	Not Clear	Malignant Vs Benign
Zhang 2 2020 [66]	Asia	Prospective	91	Imaging before biopsy	Mixed FLR and GLR	Not Clear	Malignant Vs Benign
Zhao 2018 [67]	Asia	Retrospective	1071	Imaging before biopsy	Mixed FLR and GLR	Mixed	Malignant Vs Benign
Zhi 2010 [68]	Asia	Prospective	559	Imaging before biopsy	Mixed FLR and GLR	Mixed	Malignant Vs Benign
Zhou 1 2014 [69]	Asia	Prospective	193	Imaging before biopsy	FLR	Mixed	Malignant Vs Benign
Zhou 2 2014 [69]	Asia	Prospective	193	Imaging before biopsy	GLR	Mixed	Malignant Vs Benign
Zhou 3 2014 [70]	Asia	Prospective	127	Imaging before biopsy	GLR	Mixed	Malignant Vs Benign

Key

1. *FLR* Fat to lesion ratio

2. GLR Glandular tissue to lesion ratio

3. Mixed Any combination including core needle biopsy (CNB), FNA cytology, surgical and resolution via follow up

Study distribution among continents was as follows: Asia 40 (61.54%), Europe 17 (26.15%), Africa five (7.69%), S. America two (3.08%) and N. America one (1.54%). Out of the 65 studies, their design was prospective in 51 (78.46%), retrospective 13 (20%) and one (1.54%) unclear. Regarding the year of publication, the earliest was 2007 and the latest 2020. Out of the 65 studies, 44 (67.69%) were published after 2015.

The setting in all studies was uniform in that outpatient imaging before pathologic diagnosis or treatment was the recruitment point for the participants. There were 56 (86.15%) studies that recruited only solid masses while three (4.62%) had mixed cystic and solid masses. The rest, six (9.23%) did not state the mass consistency.

Assessment of SR was carried out in comparison with other modalities in 62 (95.38%) of the studies. These were combined B-mode ultrasound (BUS) plus elastography score (ES) in 34 (52.31%), BUS eight (12.31%), ES eight (12.31%), shear wave elastography (SWE) seven (10.77%), automated strain ratio (ASR) two (3.08%), mammography one (1.54%) and magnetic resonance imaging (MRI) one (1.54%). The remaining four studies reported only results of strain elastography.

Table 1 further summarizes several variables that were derived to depict individual study characteristics. The variables included were study author identifier, continent, study design, sample size (number of lesions), setting, index test (SR measurement method), reference standard (pathological diagnosis) approach and target condition definition. A full reference to the 31 variables derived during this review is also found as an MS Excel® sheet A within the additional files.

During the reading of the eligible articles, we discovered that some had reported more than one SR measurement method for their studies. For example, an article could be having different arms that assessed the diagnostic accuracy of SR through different reference tissue points like fat-to-lesion ratio (FLR), glandular-to-lesion ratio (GLR) or even a combination of both. Thus, two or more studies would be documented from such articles, if a clear 2 × 2 table was deduced for each arm. Examples to demonstrate this include Chee 2019, Graziano 2017, Jung Park 2016, Park 2020, Yoon 2017, Zhang 2020 and Zhou 2014. These are denoted in various figures and tables with a numerical value after the author’s name.

Still, some studies did not specify the specific reference points for their measurements. FLR alone was the most used method by 38 studies (58.46%) while GLR alone was applied by five (7.69%) and a combination of both recorded in 14 (21.54%) studies. The remaining eight studies (12.31%) did not report their reference point within the scanned breast. Only four (6.15%) studies reported the lesion depth measurements. Further, 47 (72.31%) studies performed a single point SR measurement, while 10 (15.38%) did multiple points with a mean value calculated. The rest of the studies did not specify on the number of points that were used during the SR measurement.

The machine models that were used in the studies were by the following manufacturers: Hitachi 23 (35.38%), Toshiba 11 (16.92%), Philips seven (10.77%), GE Healthcare six (9.23%), Siemens four (6.15%), Samsung six (9.23%), Mindray three (4.62%) and Esaote MyLab two (3.08%). Three studies did not report the machine model that they used. There were 50 studies that reported their imaging frequency used and in the higher value, the range was between 6.5 to 15 MHz.

Experience of operators was reported in 35 (53.85%) of the studies and it ranged from 0 to 20 years.

For the size of lesions, 21 (32.31%) studies had their ranges and mean. In this subgroup, the range was from 0.1 to 13.0 cm in their longest diameter with cumulative mean diameter 1.74 cm.

Blinding to the index test while reading the reference standard was clearly stated in 59 (90.77%) studies while one reported non-blinding out of its retrospective design. The other five (7.69%) were unclear on the same.

Performance of two or more interobserver variability assessment was reported in 15 (23.08%) of the studies. Agreement was quantified and qualified in eight of the studies spanning from fair, moderate, good and excellent. The other seven just reported non-quantified agreement between the observers.

The reference standard was purely based on pathological diagnosis in majority of cases and a few incorporated follow up of indeterminate (BI-RADS III) lesions. Further, the approach to pathological diagnosis involved core biopsy, cytology and surgical (excisional biopsy or cure-intent lumpectomy) specimens. Most of the studies, 39 (60%) had a mixed approach to the reference standard. Purely core biopsy and surgical excisions were applied in 20 (30.77%) and two (3.08%) of studies, respectively. In four studies (6.15%) the reference standard approach was not stated.

All the studies had clear definition of the target condition, easily dichotomized from the pathological diagnosis. On one hand malignant lesion that would require definite aggressive treatment or intervention is defined. In the same vein, a benign lesion that would be left alone or treated based on patient symptomatology was also defined.

Timing between the index test and reference standard within six-month period was clearly reported in 26 (40%) studies while it was unclear in the rest of studies.

### Risk of bias and applicability

**Fig. 2:**
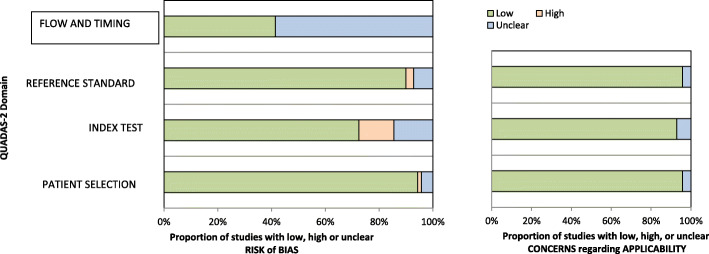
Graphic presentation of the QUADAS-2 tool assessment results. Most of the papers were of moderate to high quality. The main observation is that 41 studies (58.58%) had unclear information on the time between the index test and reference standard but the flow was acceptable in that imaging was done before pathological diagnosis

Results of the QUADAS 2 tool assessment are presented in Fig. 2. A representative table A for the individual study derivatives for the same is attached within the additional files. The papers accessed for full article reading were of moderate to high quality. Within the risk of bias 94.25, 71.43, 90.00 and 41.43% of the studies had low-risk score for patient selection, index test, standard reference and flow and timing, respectively. The main observation within the flow and timing realm, was that 58.57% of the studies had a good flow but did not succinctly indicate the time between the index test and the reference test. We interpreted this as unclear disease progression bias risk, at least theoretically.

For the applicability, in other words the generalizability of the study results, over 90% of the studies returned low-risk assessment results within the realms of the patient selection, index test and reference test. The rest were of unclear bias for example some studies subjected a specific subset of solid breast lesions to strain elastography as a problem-solving tool. Other bias points where when the point of reference in strain ratio measurement was not well stated or multiple reference standards were mentioned. As such the risks of spectrum effect, analysis bias and differential verification bias respectively were queried in this minority of studies.

## Results of individual studies

**Fig. 3:**
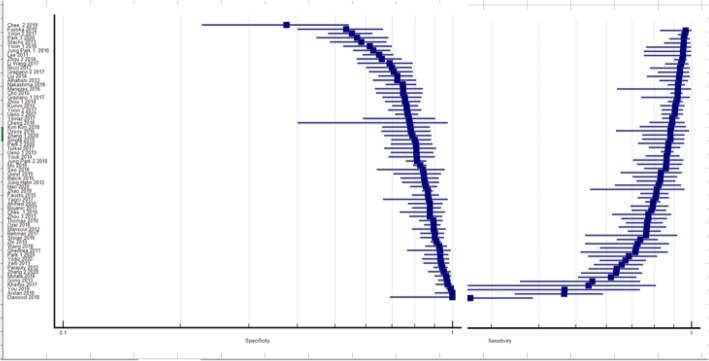
Coupled forest plot for sensitivity and specificity: studies are plotted on the y-axis. Specificity and sensitivity are on the x-axis from left to right

The detailed report of each study’s diagnostic performance in terms of 2 × 2 table derivatives such as sensitivity, specificity and odds ratios are presented in sheet A within additional files. The sensitivity values ranged from 26.47 to 96.67% and for specificity from 37.5 to 99.96%. This information is summarily presented in coupled forest plots as shown in Fig. 3. For better visualization with ability to magnify the same images are within additional files as an MS Excel® sheet B.

Index test positivity was determined using a cut-off value of the SR. In 62 (95.38%) of the studies this was derived from a ROC curve with a Youden index measure. Two (3.08%) studies were not clear on how they derived their cut-off values, while one (1.54%) study reported a predetermined vendor cut-off value.

### Heterogeneity and threshold effect

Visual inspection of the coupled forest plots of sensitivity and specificity does not reveal a smooth funnel shaped arrangement expected in good degree of homogeneity [16, 17]. This is as shown in Fig. 3 while individual forest plots for sensitivity and specificity can be accessed as figures A and B within additional files. The overall threshold effect as calculated using the Spearman correlation coefficient of Logit (TPR) vs Logit (FPR) was − 0.301 *p*-value = 0.015. A SROC plane that can be accessed within additional files as figure C, also visually demonstrates the heterogeneity. Cochran’s Q statistic values for sensitivity and specificity were 492.96 (*p* = 0.000) and 647.10 (*p =* 0.000) respectively and Higgin’s (I^2^) were 87.0 and 90.1% respectively.

### Synthesis of results and sub-group meta-analysis

Having established significant heterogeneity between the 65 studies, it was not possible to proceed to a pooled establishment of sensitivity and specificity. We delved into assessing the heterogeneities further by performing subgroup analyses. We took 13 key covariates related to participants, index test, reference standard and target condition. These were derived from the characteristics of studies worksheet. The same can be accessed within additional files as table B.

From our findings of the subgroup analyses, all covariates had heterogeneities that could not be resolved except for two that had favourable threshold effects. These were within the machine model and combination of SR with other imaging mode subgroups. The first subgroup had seven studies and the latter four studies. The four studies reported results of SR without BUS, ES or any other imaging modality. These had a borderline Spearman’s correlation coefficient of 0.600 for sensitivity vs specificity-1. A positive Spearman’s correlation coefficient value of 0.6 and above is recommended for consideration in including diagnostic accuracy studies for meta-analysis [71]. Besides, we did not consider this subgroup as a candidate for meta-analysis since the studies were too few. Again, it cannot produce a scientific basis that measurement of SR without combining with other methods would lead to a more accurate result.

**Fig. 4:**
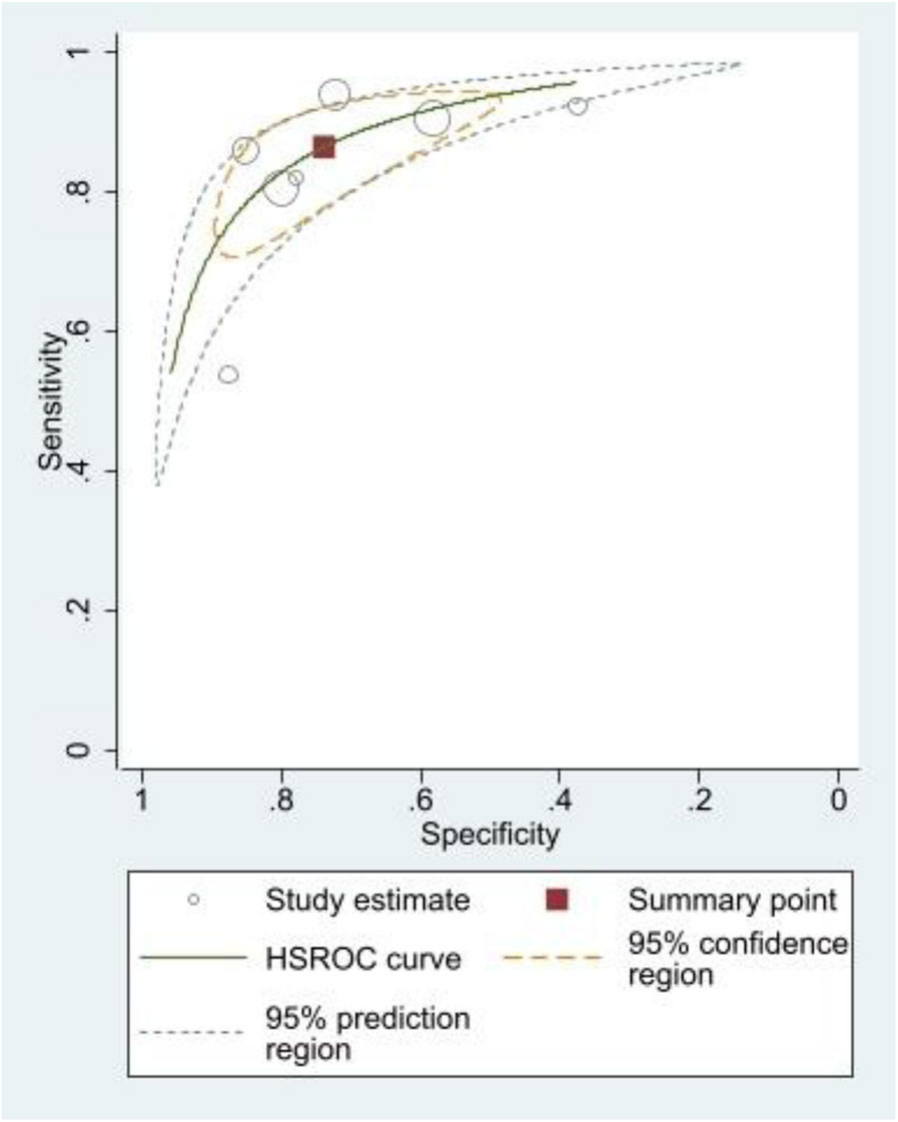
HSROC curve of seven studies within machine model subgroup that had shown favourable threshold analysis

Under machine manufacturer models covariate, we discovered one manufacturer model that produced a favourable threshold effect. This subgroup had seven studies whose sensitivity vs specificity-1 Spearman’s correlation coefficient was 0.786 (*p =* 0.036). For exploratory purposes, we carried a meta-analysis on this sub-group which consisted of 783 patients and 844 lesions. The results are presented as a hierarchical summary receiver operating characteristic curve (HSROC) in Fig. 4. Sensitivity and specificity were 0.86 and 0.74 respectively at the summary point. Using the calculated Youden index value of 0.57 the optimal cut-off value was 2.81.

### Publication bias

**Fig. 5:**
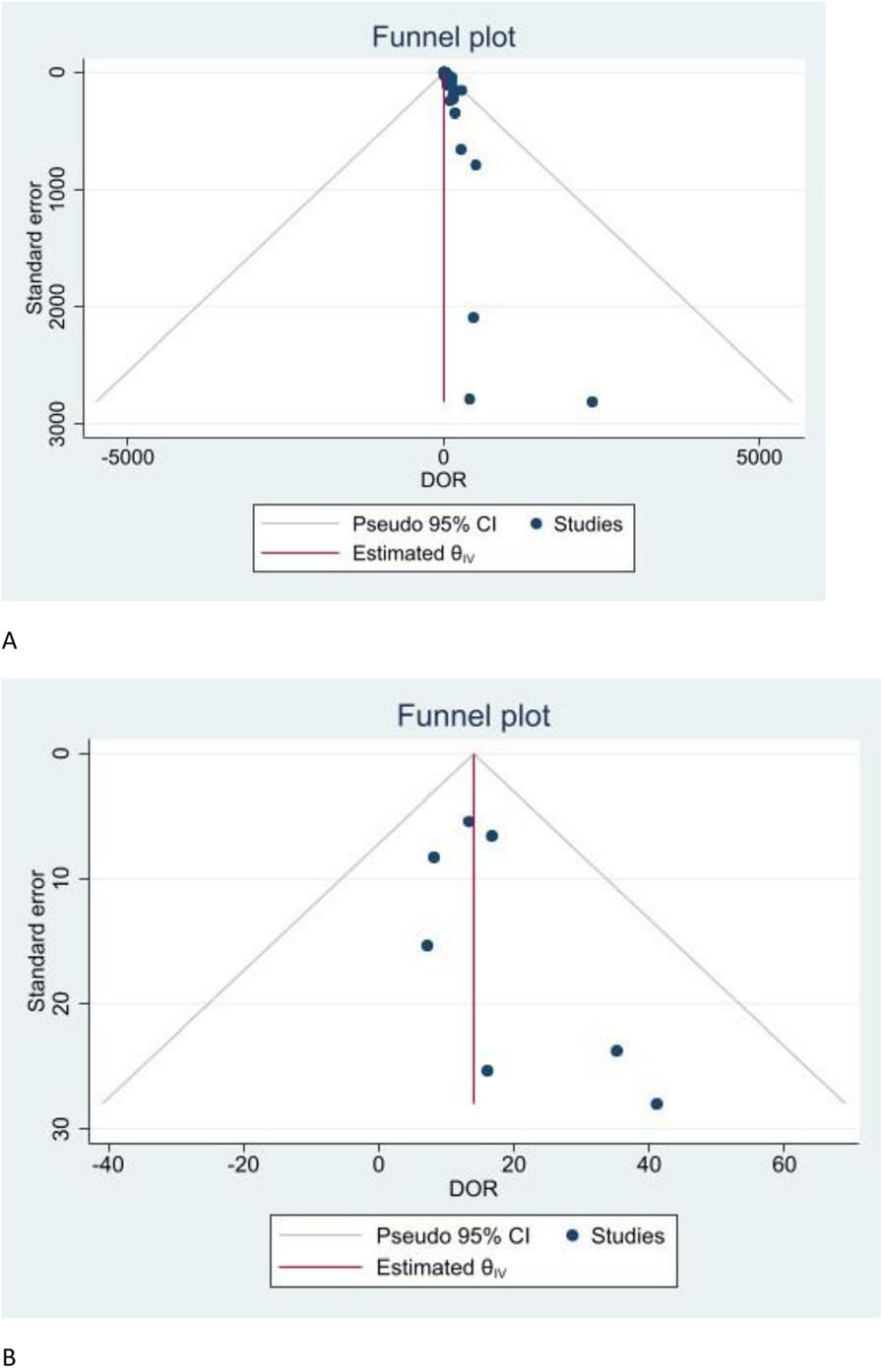
Funnel plots for a) overall studies group and b) subgroup that underwent meta-analysis

No significant publication bias was demonstrated for the 65 studies as well as for the seven that went to the sub-group meta-analysis. The funnel plots of the DORs are as shown in Fig. 5. Egger’s test was at *p*-value of 1.00 for the entire group and 0.44 for the meta-analytic subgroup.

### Additional analysis

The results of all covariate subgroup analysis demonstrating the heterogeneity factors are presented in extensive table B within additional files for reference of interest.

## Discussion

### Summary of evidence

Measurable values, either quantitative or semiquantitative in diagnostic radiology are potential imaging biomarkers that can be defined by cut-off values [72]. Breast elastography SR measurement is a semiquantitative method that we sought to establish its cut-off value for benign and malignant lesion diagnosis. From our findings during this review, breast SR measurement is still laden with multiple heterogeneities. These lie within the whole range of assessment involving the patient selection, index test, reference standard and flow and timing. The WFUMB guidelines of 2015 for breast elastography acknowledged widely varied SR cut-off values [6]. From our systematic review, nothing has changed since then.

From our results, the points of reference when carrying out the SR measurement are quite varied. This is despite the WFUMB guidelines advocating for FLR. Some researchers have been non-discriminatory on either fibro-glandular tissue or subcutaneous fat. Still, others do the ratios referencing on GLR purely. Some studies have been carried out to determine the difference of diagnostic performance dependent on FLR or GLR. We came across three studies that compared the diagnostic performance between the two points of reference by Chee et al. [19], Graziano et al. [27] and Zhou et al. [70]. They all concluded that FLR had better diagnostic performance than GLR. For general clinical application, the point of reference may have its challenges, since breast tissue composition is varied. Some breasts will be mainly fibro-glandular (dense breasts) and others almost purely fatty [73, 74]. Sorting out this point of reference heterogeneity for clinical application due to breast tissue composition requires more investigation.

Purely simple cysts are well diagnosed on B-mode ultrasound with clear designation of BI-RADS II category. Qualitative elastography also gives two characteristic appearances, the bullseye artefact or the blue-green-red (BGR) sign to cysts [75, 76]. The diagnostic value of performing SR measurements on a purely simple cyst lesion may be unnecessary when looking for an imaging biomarker that differentiates benign from malignant lesions. Benchmarking with the WFUMB guidelines, there is no clarity on the issue of SR measurement on simple cysts.

Based on its imaging functionality, SR cannot be purely used on its own without looking at the BUS and colour coded elastograms that give the elastographic score (ES). For that reason, it would be clinically meaningless to report any favourable threshold effects generated from studies that only report SR diagnostic performance.

The small subgroup of seven studies in our review that went to meta-analysis was under a particular manufacturer model. This raises a possibility that manufacturer model may influence SR values. We strongly recommend that this needs to be taken with a lot of caution as the rest of the seven machine model subgroups did not reproduce the same favourable threshold effects. Having a machine predetermined cut-off value at this stage must  depend on more robust evidence. In fact, the only study in this review that reported using a vendor machine predetermined cut-off value fell in a subgroup that did not produce favourable threshold effects. Furthermore, there are pieces of literature that indicate quantified variance for in vitro and clinical applications of SR measurement [77, 78]. We still propose more research on the manufacturer model calibration aspects.

Operator dependency in ultrasound is a fact that may affect semiquantitative procedures like SR [78]. It is not an easily quantifiable entity even though we explored through some surrogates like experience of operators as well as studies that conducted interobserver variability assessment. None of those on their own could produce a favourable threshold effect.

Our review had two limitations. First, we could not have access to Embase, one of the most renown electronic databases due to lack of institutional accessibility at the time of conducting our search. Despite this we did the best in the search as per published evidence as we accessed three of four major databases good enough for a systematic review [79]. On this front, we remain open to more forthcoming evidence concerning our topic in the future. Another limitation is that efforts to get more information from some authors whom we contacted for certain clarifications were not replied to.

## Conclusions

From our review, currently the optimal breast SR cut-off point or value remains unresolved despite the WFUMB guidelines of 2015. Machine model possibility as a contributor to cut-off value determination was suggested from this review which can be subjected to more industry and multi-center research determination.

## Supplementary Information

**Additional file 1.**

**Additional file 2.**

**Additional file 3.**

**Additional file 4.**

**Additional file 5.**

**Additional file 6.**

## Data Availability

Raw data of variables obtained from individual studies is found within the additional (supplementary) files herein attached. Any further information on datasets can be provided by the corresponding author on considerable request.

## Abbreviations

ASR: Automated strain ratio
BI-RADS: Breast Imaging-Reporting and Data System
BUS: B-mode ultrasound
DOR: Diagnostic odds ratio
ES: Elastography score
FLR: Fat-to-lesion ratio
FPR: False positive rate
GLR: Glandular-to-lesion ratio
HSROC: Hierarchical summary receiver operating characteristic
MRI: Magnetic resonance imaging
NLR: Negative likelihood ratio
PLR: Positive likelihood ratio
PRISMA-DTA: Preferred Reporting Items for Systematic Reviews and Meta-Analyses for Diagnostic Test Accuracy
QUADAS: Quality Assessment of Diagnostic Accuracy Studies
STARD: Standards for Reporting of Diagnostic Accuracy Studies
SR: Strain ratio
SROC: Summary receiver operating characteristic
TPR: True positive rate
WFUMB: World Federation for Ultrasound in Medicine and Biology
