# Supplementary figures and images for: Determining the elastography strain ratio cut off value for differentiating benign from malignant breast lesions: systematic review and meta-analysis
Source: Cancer Imaging. 2022 Feb 12;22:12. doi: 10.1186/s40644-022-00447-5 (PMC8841096; doi:10.1186/s40644-022-00447-5)

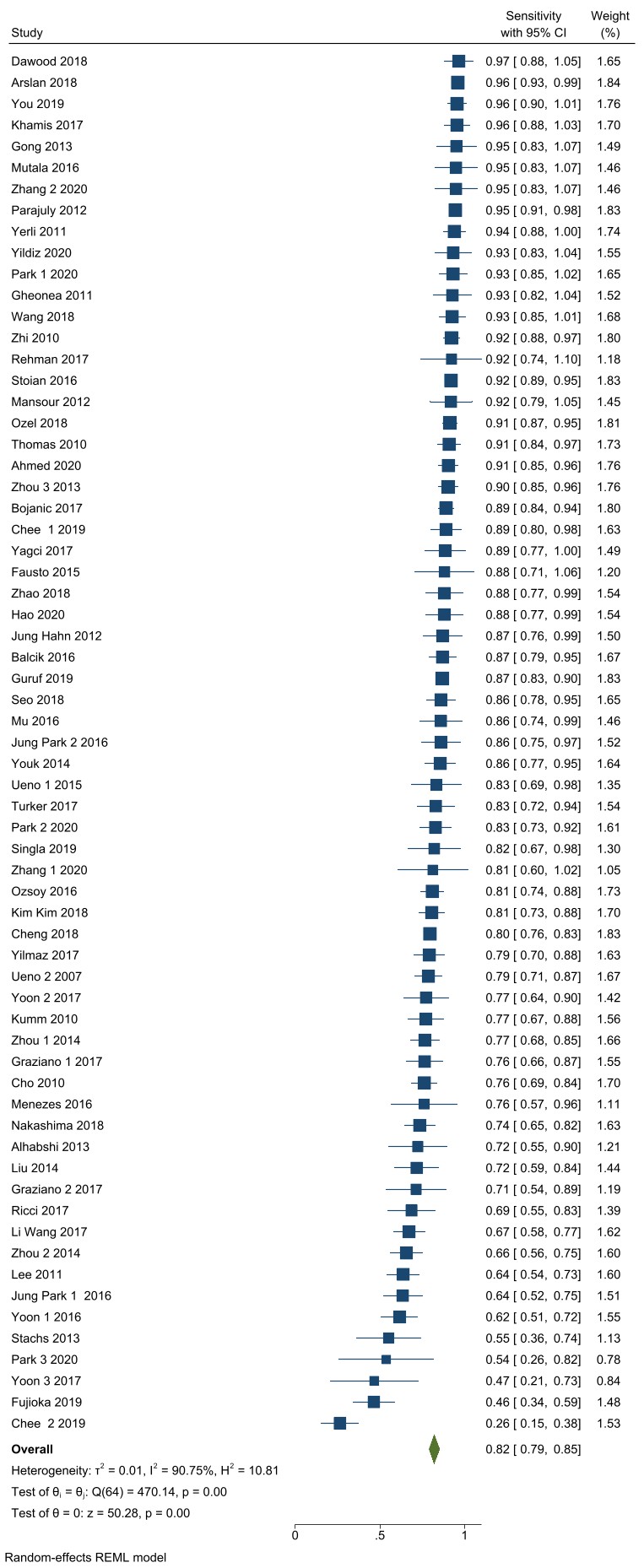

Supplement: Supplementary file 1 — Additional file 1. [file 40644_2022_447_MOESM1_ESM.jpg]

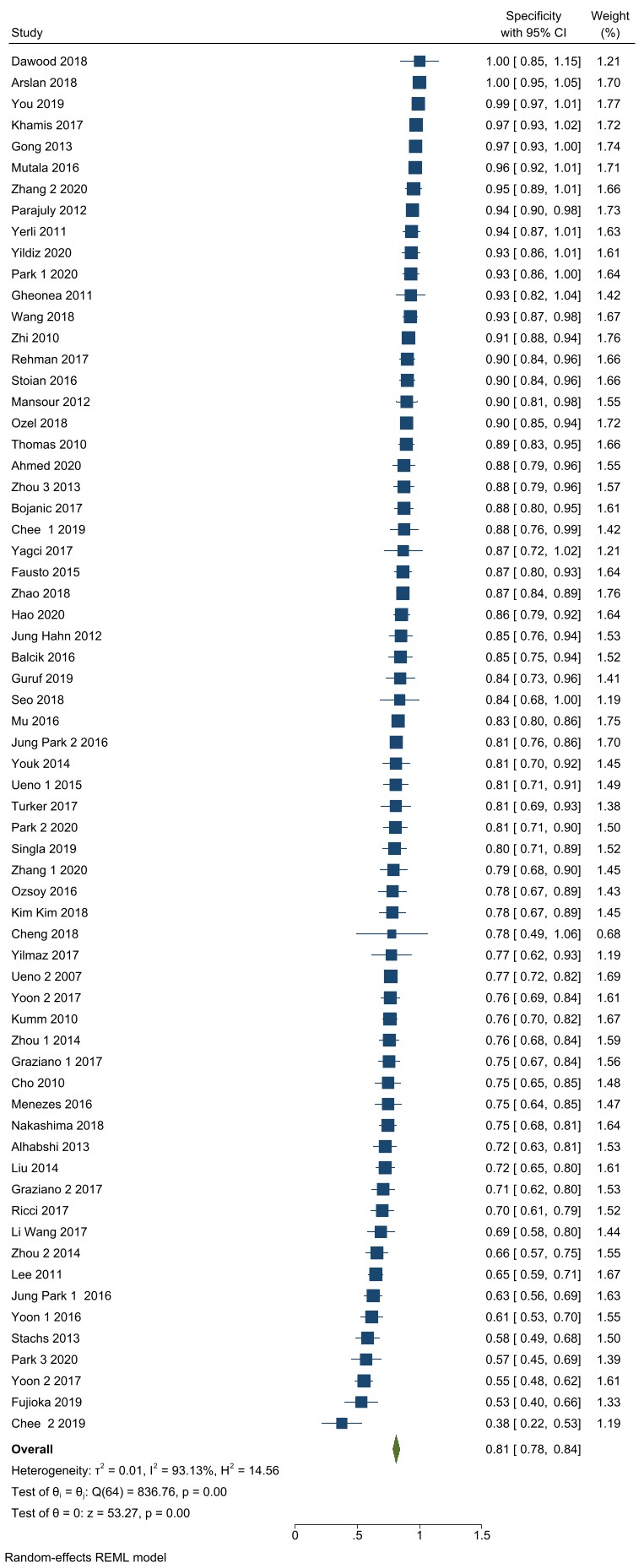

Supplement: Supplementary file 2 — Additional file 2. [file 40644_2022_447_MOESM2_ESM.jpg]

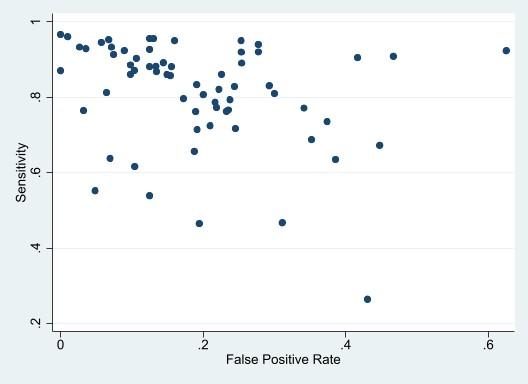

Supplement: Supplementary file 3 — Additional file 3. [file 40644_2022_447_MOESM3_ESM.jpg]
